# Frequency, Antimicrobial Resistance and Genetic Diversity of *Klebsiella pneumoniae* in Food Samples

**DOI:** 10.1371/journal.pone.0153561

**Published:** 2016-04-14

**Authors:** Yumei Guo, Haijian Zhou, Liyun Qin, Zhizhao Pang, Tian Qin, Hongyu Ren, Zhuo Pan, Jikun Zhou

**Affiliations:** 1 Shijiazhuang Center for Disease Control and Prevention, Shijiazhuang, People’s Republic of China; 2 State Key Laboratory for Infectious Disease Prevention and Control, National Institute for Communicable Disease Control and Prevention, Chinese Center for Disease Control and Prevention, Beijing, People’s Republic of China; 3 Collaborative Innovation Center for Diagnosis and Treatment of Infectious Diseases, Hangzhou, People’s Republic of China; Second University of Naples, ITALY

## Abstract

This study aimed to assess the frequency of *Klebsiella pneumoniae* in food samples and to detect antibiotic resistance phenotypes, antimicrobial resistance genes and the molecular subtypes of the recovered isolates. A total of 998 food samples were collected, and 99 (9.9%) *K*. *pneumoniae* strains were isolated; the frequencies were 8.2% (4/49) in fresh raw seafood, 13.8% (26/188) in fresh raw chicken, 11.4% (34/297) in frozen raw food and 7.5% (35/464) in cooked food samples. Antimicrobial resistance was observed against 16 antimicrobials. The highest resistance rate was observed for ampicillin (92.3%), followed by tetracycline (31.3%), trimethoprim-sulfamethoxazole (18.2%), and chloramphenicol (10.1%). Two *K*. *pneumoniae* strains were identified as extended-spectrum β-lactamase (ESBL)–one strain had three beta-lactamases genes (*bla*_SHV_, *bla*_CTX-M-1_, *and bla*_CTX-M-10_) and one had only the *bla*_SHV_ gene. Nineteen multidrug-resistant (MDR) strains were detected; the percentage of MDR strains in fresh raw chicken samples was significantly higher than in other sample types (P<0.05). Six of the 18 trimethoprim-sulfamethoxazole-resistant strains carried the folate pathway inhibitor gene (*dhfr*). Four isolates were screened by PCR for quinolone resistance genes; *aac(6’)-Ib-cr*, *qnrB*, *qnrA* and *qnrS* were detected. In addition, *gyrA* gene mutations such as T247A (Ser83Ile), C248T (Ser83Phe), and A260C (Asp87Ala) and a *parC* C240T (Ser80Ile) mutation were identified. Five isolates were screened for aminoglycosides resistance genes; *aacA4*, *aacC2*, and *aadA1* were detected. Pulsed-field gel electrophoresis-based subtyping identified 91 different patterns. Our results indicate that food, especially fresh raw chicken, is a reservoir of antimicrobial-resistant *K*. *pneumoniae*, and the potential health risks posed by such strains should not be underestimated. Our results demonstrated high prevalence, antibiotic resistance rate and genetic diversity of *K*. *pneumoniae* in food in China. Improved control and prevention strategies are urgently needed.

## Introduction

*Klebsiella pneumoniae* is a common opportunistic pathogen that causes human infections. It can be widely distributed not only in the respiratory and intestinal tracts of humans and animals but also in a variety of environments and vectors. This pathogen can cause pneumonia, respiratory tract infections, urinary system infections, septicemia and other diseases [1,2]. Antimicrobials have been widely used to treat *K*. *pneumoniae* infections in humans. However, increasing antimicrobial resistance, especially that mediated by extended-spectrum β-lactamases (ESBL), plasmid-borne AmpCs, and carbapenemases, has been reported in recent years and has become a serious problem [3–5].

Foodborne diseases caused by pathogenic bacteria constitute a serious threat to public health worldwide [6,7]. Until now, most investigations on foodborne bacteria focused on common foodborne pathogens, such as *Salmonella*, *Campylobacter*, *Escherichia coli*, *Shigella*, *Listeria monocytogenes*, *Staphylococcus aureus*, and *Vibrio parahaemolyticus*. In contrast, little information was obtained on foodborne *K*. *pneumoniae* as *K*. *pneumoniae* is generally not recognized as a foodborne pathogen. However, antimicrobial-resistant *K*. *pneumoniae* strains have been isolated from marketed fresh vegetables [8], shrimp in international trade [9], and farm-raised chicken [10]. A recent report showed that foodborne *K*. *pneumoniae* could cause a nosocomial outbreak [11]. Furthermore, several resistance genes in *K*. *pneumoniae* are located in transferable genetic elements that may be transferred to other bacteria. Thus, the potential contribution of *K*. *pneumoniae* to the resistance of clinically relevant bacteria is cause for concern.

The presence of antimicrobial-resistant *K*. *pneumoniae* strains in the food supply is alarming. Our objective was to assess the frequency of *K*. *pneumoniae* in food samples. We focused on the contamination rate in foods and on the characteristics of *K*. *pneumoniae* isolates. We characterized their antimicrobial resistance phenotypes, identified their antimicrobial resistance genes and analyzed their molecular subtypes.

## Materials and Methods

### Sample collection

A total of 998 food samples were collected in Shijiazhuang, a city of approximately 10 million inhabitants in eastern China, between April 2013 and July 2014. Those samples were used to isolate *K*. *pneumoniae* strains. The samples included 49 fresh raw seafood (fish, shellfish, shrimp) samples, 188 fresh raw chicken samples, 297 frozen raw food (meat, vegetables, flour and rice products) samples and 464 cooked food samples (meat, vegetables, flour and rice products). These samples were collected from different farms, supermarkets and restaurants distributed throughout the city. None of the samples were duplicated.

### Isolation and identification

A 25-g portion of each sample was suspended in 225 mL of buffered peptone water (BPW). The sample suspensions were incubated overnight at 36°C. A 1-mL aliquot of the pre-enrichment culture was added to 10 mL of selenite cystine broth (SC) and incubated overnight at 36°C. A loopful (10 μL) of SC was streaked directly onto Salmonella Shigella (SS) agar plates and incubated for 24 h at 36°C. Colorless, medium-sized, smooth and moist colonies were transferred to triple-sugar iron (TSI) agar plates. All the suspected *K*. *pneumoniae* isolates were identified using a BD Phoenix™-100 Automated Microbiology System (Becton, Dickinson and Company, Sparks, Maryland, USA).

### Antimicrobial susceptibility testing

Antimicrobial susceptibility testing for the *K*. *pneumoniae* strains was performed using a BD Phoenix NMIC/ID-4 system according to the manufacturer’s instructions. The following 21 antimicrobials were tested: amikacin (AMI), gentamicin (GEN), imipenem (IPM), meropenem (MEM), cefazolin (CZO), ceftazidime (CAZ), cefotaxime (CTX), cefepime (FEP), aztreonam (ATM), ampicillin (AMP), piperacillin (PRL), amoxicillin-clavulanate (AMC), ampicillin-sulbactam (SAM), piperacillin-tazobactam (TZP), colistin (CL), trimethoprim-sulfamethoxazole (SXT), chloramphenicol (C), ciprofloxacin (CIP), levofloxacin (LVX), moxifloxacin (MXF), and tetracycline (TE). The minimum inhibitory concentrations (MICs) were interpreted by the standards of Clinical and Laboratory Standards Institute (CLSI) document M100-S24:2014 [12]. The presence of ESBLs was detected with the BD Phoneix NMIC/ID-4 test and was further confirmed by the double-disk diffusion method [12]. *Escherichia coli* strain ATCC 25922 and *K*. *pneumoniae* strain ATCC 700603 were used as quality-control strains for the antimicrobial susceptibility testing.

A standardized international definition was used to define multidrug-resistant (MDR) bacteria [13]. MDR was defined as acquired non-susceptibility to at least 1 agent in 3 or more antimicrobial categories.

### PCR amplification and sequencing

Genomic DNA was extracted using a QIAamp DNA minikit (Qiagen, Dusseldorf, Germany) or prepared by the boiling method. Antimicrobial resistance-associated genes were detected by PCR and sequenced using the primers listed in Table 1. The PCR was performed in a 50-μL reaction volume that contained 25 μL of Premix Taq ^TM^ (Takara, Dalian, China), 10 μM of each primer and 1 μL of sample DNA. The PCR conditions for the β-lactamase genes consisted of an initial denaturation at 95°C for 5 min, 35 cycles of denaturation at 95°C for 50 s, annealing at 56°C, 50°C or 60°C for 40 s and elongation at 72°C for 1 min, followed by a final extension at 72°C for 5 min, in a thermocycler (Labcycler, Senso, Germany). The PCR conditions for other resistance genes consisted of an initial denaturation at 95°C for 5 min, 30 cycles of denaturation at 94°C for 1 min, annealing at 55°C for 1 min and elongation at 72°C for 2 min, followed by a final extension at 72°C for 5 min, in a thermocycler. The PCR products were detected in a 1% agarose gel. Positive amplicons were sequenced on a PE Applied Biosystems ABI Prism 3730 instrument. The DNA sequences were annotated using the BLAST program (http://blast.ncbi.nlm.nih.gov) to identify the gene subtypes. Mutations in the *gyrA* and *parC* sequences of *K*. *pneumoniae* (reference GenBank accession numbers DQ673325 and NC009648 for *gyrA* and *parC*, respectively) were detected.

**Table 1:** Primers Used for PCR Amplification and Resistance Gene Sequencing.

Gene	Primer Sequence (5’→3’)		Annealing Temp (°C)	Fragment (bp)	Reference
	Forward	Reverse						
**β - lactamase genes**					
*bla*_TEM_	TCAACATTTCCGTGTCG	CTGACAGTTACCAATGCTTA	56	860	[14]
*bla*_SHV_	ATGCGTTATATTCGCCTGTG	AGATAAATCACCACAATGCGC	56	896	[14]
*bla*_CTX-M-1_	CCGTTTCCGCTATTACAAACCG	GGCCCATGGTTAAAAAATCACTGC	56	944	[15]
*bla*_CTX-M-2_	ATGATGACTCACAGCATTCG	TCCCGACGGCTTTCCGCGTT	56	833	[16]
*bla*_CTX-M-8_	TTTGCCCGTGCGATTGG	CGACTTTCTGCCTTCTGCTCT	50	368	[17]
*bla*_CTX-M-9_	ATGGTGACAAAGAGAGTGCA	CCCTTCGGCGATGATTCTC	50	870	[18]
*bla*_CTX-M-10_	GCAGCACCAGTAAAGTGATGG	GCGATATCGTTGGTGGTACC	56	524	[19]
*bla*_CTX-M-14_	GAGAGTGCAACGGATGATG	TGCGGCTGGGTAAAATAG	56	941	[20]
**AmpC genes**					
*ba*_CMY-G1_	GCTGACAGCCTCTTTCTCCAC	CCTCGACACGGRCAGGGTTA	56	1082	[21]
*ba*_CMY-G2_	GGTCTGGCCCATGCAGGTGA	GGTCGAGCCGGTCTTGTTGA	56	963	[21]
*bla*_DHA_	AACTTTCACAGGTGTGCTGGGT	CCGTACGCATACTGGCTTTGC	60	405	[22]
*bla*_ACT_	ATTCGTATGCTGGATCTCGCCACC	CATGACCCAGTTCGCCATATCCTG	50	396	[23]
*bla*_FOX_	CACCACGAGAATAACC	GCCTTGAACTCGACCG	50	1184	[23]
**Folate pathway inhibitors**					
*dhfr*	GCCAATCGGGTTATTGGCAA	TGGGAAGAAGGCGTCACCCTC	55	357	[24]
**Fluoroquinolone resistance-associated genes**					
*qnrA*	ATTTCTCACGCCAGGATTTG	GATCGGCAAAGGTTAGGTCA	55	627	[25]
*qnrB*	GATCGTGAAAGCCAGAAAGG	ACGATGCCTGGTAGTTGTCC	55	469	[25]
*qnrC*	GGGTTGTACATTTATTGAATCG	CACCTACCCATTTATTTTCA	55	307	[26]
*qnrD*	CGAGATCAATTTACGGGGAATA	AACAAGCTGAAGCGCCTG	55	533	[27]
*qnrS*	ACGACATTCGTCAACTGCAA	TAAATTGGCACCCTGTAGGC	55	417	[28]
*aac(6’)-Ib-cr*	TTGCGATGCTCTATGAGTGGCTA	CTCGAATGCCTGGCGTGTTT	55	482	[29]
*qepA*	AACTGCTTGAGCCCGTAGAT	GTCTACGCCATGGACCTCAC	55	596	[26]
*gyrA*	CGACCTTGCGAGAGAAAT	GTTCCATCAGCCCTTCAA	55	626	[30]
*parC*	TACGTCATCATGGACAGG	GCCACTTCACGCAGGTTG	55	460	[31]
**Aminoglycoside resistance-associated genes**					
*aacA4*	ATGACTGA CATGACCTTGCG	TTAGGCATCACTGCGTGTTCG	55	540	[32]
*aacC1*	ATGGGCATCATTCGCACATGTAGG	TTAGGTGGCGGTACTTGGGTC	55	873	[32]
*aacC2*	ATGCATACGCGGAAGGCAATAAC	CTAACCGGAAGGCTCGCAAG	55	861	[32]
*aadA1*	ATGAGGGAAGCGGTGATCG	TTATTTGCCGACTACCTTGGTG	55	792	[32]
*aadB*	ATGGACACAACGCAGGTCGC	TTAGGCCGCATATCGCGACC	55	534	[32]
*aphA6*	ATGGAATTGCCCAATATTATTC	TCAATTCAATTCATCAAGTTTTA	55	781	[32]
*armA*	AGGTTGTTTCCATTTCTGAG	TCTCTTCATTCCCTTCTCC	55	591	[33]
*rmtB*	CCCAAACAGACCGTAGAGGC	CTCAAACTCGGCGGGCAAGC	55	585	[33]
Integron I	GGCATCCAAGCACAAG	AAGCAGACTTGACCTGA	55	Variable	[34]

### Pulsed-field gel electrophoresis (PFGE)

We used the 1-day, standardized PFGE protocol for *K*. *pneumoniae* [35]. Cell suspensions were placed in polystyrene tubes (Falcon; 12 × 75 mm), and their optical densities were adjusted to 3.8–4.0 using a Densimat photometer (BioMérieux, Marcy l’Etoile, France). Slices of *K*. *pneumoniae* agarose plugs were digested using 50 U of *Xba*I (Takara) per slice for 4 h at 37°C, and electrophoresis was performed using a CHEF-DRIII system (Bio-Rad Laboratories, Hercules, CA, USA). Electrophoresis was conducted with a switch time of 6 s to 36 s for 18.5 h, and images were captured using a Gel Doc 2000 system (Bio-Rad) and converted to TIFF files. The TIFF files were analyzed using BioNumerics version 5.1 software (Applied Maths, Kortrijk, Belgium). A similarity analysis of the PFGE patterns was performed by calculating the Dice coefficients (SD) [36] and clustering was performed using the unweighted-pair group method with average linkages (UPGMA).

### Multilocus sequence typing (MLST)

MLST with 7 genes (*gapA*, *infB*, *mdh*, *pgi*, *phoE*, *rpoB* and *tonB*) was performed on the isolates as previously described [37]. Alleles and sequence types (STs) were assigned using the *K*. *pneumoniae* MLST database (http://bigsdb.web.pasteur.fr/klebsiella/klebsiella.html).

### Statistical analysis

SPSS software (version 15.0) was used to statistically analyze the data. Categorical variables were compared using the Fisher’s exact test. A P value <0.05 was considered to be statistically significant.

## Results

### Contamination rate of food samples with *K*. *pneumoniae*

In total, 998 food samples were tested in this study, and *K*. *pneumoniae* was cultured from 99 of those samples. Overall, 9.9% of the food samples were positive for *K*. *pneumoniae*. *K*. *pneumoniae* was cultured from 8.2% (4/49) of the fresh raw seafood samples, 13.8% (26/188) of the fresh raw chicken samples, 11.4% (34/297) of the frozen raw food samples and 7.5% (35/464) of the cooked food samples. The rates of *K*. *pneumoniae* isolation among sample types were significantly different (Fisher’s exact test, P<0.05). In total, 31, 33, and 35 strains were isolated from the food raw materials, processing, and marketing sectors, respectively.

### Antimicrobial susceptibility patterns of the *K*. *pneumoniae* isolates

Antimicrobial susceptibility testing was conducted for the 99 *K*. *pneumoniae* isolates, and detailed information on the resistance rates to all of the tested antimicrobials is listed in Table 2. The highest resistance rate was observed for AMP, which reached 92.3% (n = 92), followed by resistance to TE (n = 31; 31.3%), SXT (n = 18; 18.2%), C (n = 10; 10.1%), and 12 other antimicrobials with resistance rates under 10.0%. There was no resistance noted to carbapenems (IPM, MEM). Notably, the resistances to 7 antimicrobials (GEN, CTX, FEP, ATM, SAM, CIP, or LVX) were detected only among fresh raw chicken isolates. Furthermore, the rate of resistance to 5 antimicrobials (CZO, PRL, SXT, C and TE) in fresh raw chicken isolates was significantly higher than in isolates from other types of samples (P<0.05). Two *K*. *pneumoniae* strains were detected as ESBL-producing; both were from fresh raw chicken samples.

**Table 2:** Antimicrobial Resistance Rates of 99 *K*. *pneumoniae* Isolates.

Antimicrobial category	Antimicrobial	Range (μg/mL)	susceptible MIC	Intermediate MIC	Resistant MIC	Raw seafood isolates (n = 4)	Raw chicken isolates (n = 26)	Frozen raw food isolates (n = 34)	Cooked food isolates (n = 35)	Total
						R (%)	R (%)	R (%)	R (%)	R (%)
**Aminoglycosides**	Amikacin	8–32	≤8		≥32	0	0	0	0	0
	Gentamicin	2–8	≤2	4	>8	0	5 (19.2%)	0	0	5 (5.1%)
**Carbapenems**	Imipenem	1–8	≤8		>8	0	0	0	0	0
	Meropenem	1–8	≤8		>8	0	0	0	0	0
**1**^**st**^**-generation cephalosporins**	Cefazolin	4–16	≤4	16	>16	0	2 (7.7%)	2 (5.9%)	0	4 (4.0%)
**3**^**rd**^**- and 4**^**th**^**-generation cephalosporins**	Ceftazidime	1–16	≤1	2	>16	0	0	0	0	0
	Cefotaxime	1–32	≤1	2	>32	0	1 (3.8%)	0	0	1 (1.0%)
	Cefepime	2–16	≤2		>16	0	1 (3.8%)	0	0	1 (1.0%)
**Monobactams**	Aztreonam	2–16	≤2	16	>16	0	0	0	0	0
**Penicillins**	Ampicillin	4–16	≤4–8	16	>16	4 (100%)	25 (96.2%)	31 (91.2%)	32 (91.4%)	92 (92.9%)
	Piperacillin	4–64	≤4–16	32	>64	0	3 (11.5%)	0	1 (2.9%)	4 (4.0%)
**Antipseudomonal penicillins+β-lactamase inhibitors**	Amoxicillin-Clavulanate	4/2-16/8	≤4/2-8/4	16/8	>16/8	0	2 (7.7%)	1 (2.9%)	0	3 (3.0%)
	Ampicillin-Sulbactam	4/2-16/8	≤4/2-8/4	16/8	>16/8	0	4(21.4%)	0	0	4 (4.0%)
	Piperacillin-Tazobactam	4/4-64/4	≤4/4	8/4-16/4	>64/4	0	0	0	0	0
**Others**	Colistin	0.5–2	≤0.5	1	>2	0	0	0	0	0
**Folate pathway inhibitors**	Trimethoprim-Sulfamethoxazole	0.5/9.5-2/38	≤0.5/9.5	1/19	>2/38	0	13 (50.0%)	2 (5.9%)	3 (8.6%)	18 (18.2%)
**Chloramphenicols**	Chloramphenicol	4–16	≤4	8–16	>16	0	8 (30.8%)	1 (2.9%)	1 (2.9%)	10 (10.1%)
**Fluoroquinolones**	Ciprofloxacin	0.5–2	≤0.5–1	2	>2	0	6 (23.1%)	0	0	6 (5.9%)
	Levofloxacin	1–8	≤1		>8	0	3 (11.5%)	0	0	3 (3.0%)
	Moxifloxacin	1–4	≤1	2–4	>4	0	0	0	0	0
**Tetracycline antibiotics**	Tetracycline	2–8	≤2		>8	0	21 (80.8%)	4 (11.8%)	6 (17.1%)	31 (31.3%)

Nineteen MDR strains were detected among 99 *K*. *pneumoniae* isolates. The proportions of MDR strains in different samples were 50.0% (14/28), 11.4% (4/35), 2.9% (1/34) and 0% (0/4), in fresh raw chicken, cooked food samples, frozen raw food and fresh raw seafood, respectively. The proportion of MDR isolates from fresh raw chicken samples was significantly higher than that from other types of samples (P<0.05).

### Antimicrobial resistance determinants of the *K*. *pneumoniae* isolates

According to the results of antimicrobial susceptibility testing, 2, 16, 4 and 5 strains were selected to analyze ESBL genes, folate pathway inhibitor genes, fluoroquinolone resistance genes and aminoglycoside resistance genes, respectively. For the 2 ESBL strains, 8 β-lactamase genes and 5 AmpC genes were amplified. As shown in Table 3, 1 strain carried *bla*_SHV_, *bla*_CTX-M-1_ and *bla*_CTX-M-10_, and the other carried *bla*_SHV_. No *bla*_CTX-M-9_, *bla*_CTX-M-14_, *bla*_DHA_, *bla*_TEM_, *ba*_CMY_, *bla*_ACT_, or *bla*_FOX_ genes were detected in these isolates.

**Table 3:** Characteristics of the 2 ESBL-Producing *K*. *pneumoniae* Isolates Detected in this Study.

Strain ID	Antimicrobial resistance patterns ^a^	ESBL genes	MLST type
SJZ2013N33	GEN-CZO-CAZ-CTX-FEP-ATM-AMP-PRL-AMC-SAM-SXT-C-TE	SHV, CTX-M-1, CTX-M-10	1651
SJZ2013N75	GEN-CZO-CAZ-CTX-FEP-ATM-AMP-PRL-AMC-SAM-SXT-C-TE	SHV	1652

^a^ Abbreviations of antimicrobials: AMI, amikacin; GEN, gentamicin; IPM, imipenem; MEM, meropenem; CZO, cefazolin; CAZ, ceftazidime; CTX, cefotaxime; FEP, cefepime; ATM, aztreonam; AMP, ampicillin; PRL, piperacillin; AMC, amoxicillin-clavulanate; SAM, ampicillin-sulbactam; TZP, piperacillin-tazobactam; CL, colistin; SXT, trimethoprim-sulfamethoxazole; C, chloramphenicol; CIP, ciprofloxacin; LVX, levofloxacin; MXF, moxifloxacin; TE, tetracycline.

Eighteen isolates that showed trimethoprim-sulfamethoxazole resistance were selected for folate pathway inhibitor gene (*dhfr*) testing; 6 of the isolates were positive for *dhfr*. All of the 6 *dhfr-*positive isolates were isolated from fresh raw chicken, whereas no isolates from frozen raw food or cooked food samples tested were positive for *dhfr*.

Four isolates were tested for fluoroquinolone resistance determinants. Among the 7 plasmid-encoded fluoroquinolone resistance-associated genes analyzed in this study, namely *qnrA*, *qnrB*, *qnrC*, *qnrD*, *qnrS*, *aac(6’)-Ib-cr*, and *qepA*, 4 genes were detected (Table 4). Among the 4 tested isolates, *aac(6’)-Ib-cr*, *qnrB*, *qnrA* and *qnrS* were detected in 4, 2, 1 and 1 isolate(s), respectively. In addition, *gyrA* gene mutations, such as T247A (Ser83Ile; two isolates), C248T (Ser83Phe; one isolate), and A260C (Asp87Ala; one isolate), and the *parC* gene mutation C240T (Ser80Ile; one isolate), were identified.

**Table 4:** Characteristics of the Fluoroquinolone Resistance-Associated Genes in 4 Fluoroquinolone-Resistant or Intermediately Fluoroquinolone-Resistant *K*. *pneumoniae* Isolates Detected in this Study.

Strain ID	Antimicrobial resistance patterns ^b^	Fluoroquinolone resistance-associated genes	*gyrA* mutation
SJZ2013N75 ^a^	GEN-CZO-CAZ-CTX-FEP-ATM-AMP-PRL-AMC-SAM-SXT-C-TE	*qnrB*, *aac(6’)-Ib-cr*	
SJZ2013N28	AMP-PRL-SXT-C-CIP-TE	*qnrB*, *aac(6’)-Ib-cr*	
SJZ2013N70	GEN-AMP-SAM-SXT-C-CIP-LVX-TE	*qnrS*, *aac(6’)-Ib-cr*	T247A (Ser83Ile)
SJZ2013N7	GEN-AMP-SAM-C-CIP-LVX-TE	*qnrA*, *aac(6’)-Ib-cr*	

^a^ SJZ2013N75 showed intermediate resistance to CIP.

^b^ Abbreviations of antimicrobials: AMI, amikacin; GEN, gentamicin; IPM, imipenem; MEM, meropenem; CZO, cefazolin; CAZ, ceftazidime; CTX, cefotaxime; FEP, cefepime; ATM, aztreonam; AMP, ampicillin; PRL, piperacillin; AMC, amoxicillin-clavulanate; SAM, ampicillin-sulbactam; TZP, piperacillin-tazobactam; CL, colistin; SXT, trimethoprim-sulfamethoxazole; C, chloramphenicol; CIP, ciprofloxacin; LVX, levofloxacin; MXF, moxifloxacin; TE, tetracycline.

Among the aminoglycoside resistance-associated genes, the *aacA4*, *aacC2*, *aadA1* genes were detected in 4, 3 and 1 isolate(s), respectively (Table 5). No *aacC1*, *aadB*, *aphA6*, *armA*, *rmtB* or Integron I genes were detected in this study. Among the 5 tested isolates, 3 isolates carried both *aacA4* and *aacC2*; 1 isolate carried both *aacA4* and *aadA1*, and 1 isolate carried none of these genes.

**Table 5:** Characteristics of the Aminoglycoside Resistance-Associated Genes in 5 Gentamicin-Resistant or Intermediately Gentamicin-Resistant *K*. *pneumoniae* Isolates Detected in this Study.

Strain ID	Antimicrobial resistance patterns ^a^	aminoglycoside resistance-associated genes
SJZ2013N33	GEN-CZO-CAZ-CTX-FEP-ATM-AMP-PRL-AMC-SAM-SXT-C-TE	*aacA4*, *aacC2*
SJZ2013N75	GEN-CZO-CAZ-CTX-FEP-ATM-AMP-PRL-AMC-SAM-SXT-C-TE	*aacA4*, *aacC2*
SJZ2013N70	GEN-AMP-SAM-SXT-C-CIP-LVX-TE	*aacA4*, *aadA1*
SJZ2013N7	GEN-AMP-SAM-C-CIP-LVX-TE	*aacA4*, *aacC2*
SJZ2013N4	GEN-AMP-C	

^a^ Abbreviations of antimicrobials: AMI, amikacin; GEN, gentamicin; IPM, imipenem; MEM, meropenem; CZO, cefazolin; CAZ, ceftazidime; CTX, cefotaxime; FEP, cefepime; ATM, aztreonam; AMP, ampicillin; PRL, piperacillin; AMC, amoxicillin-clavulanate; SAM, ampicillin-sulbactam; TZP, piperacillin-tazobactam; CL, colistin; SXT, trimethoprim-sulfamethoxazole; C, chloramphenicol; CIP, ciprofloxacin; LVX, levofloxacin; MXF, moxifloxacin; TE, tetracycline.

### PFGE and MLST analysis of *K*. *pneumoniae* isolates

**Fig 1:**
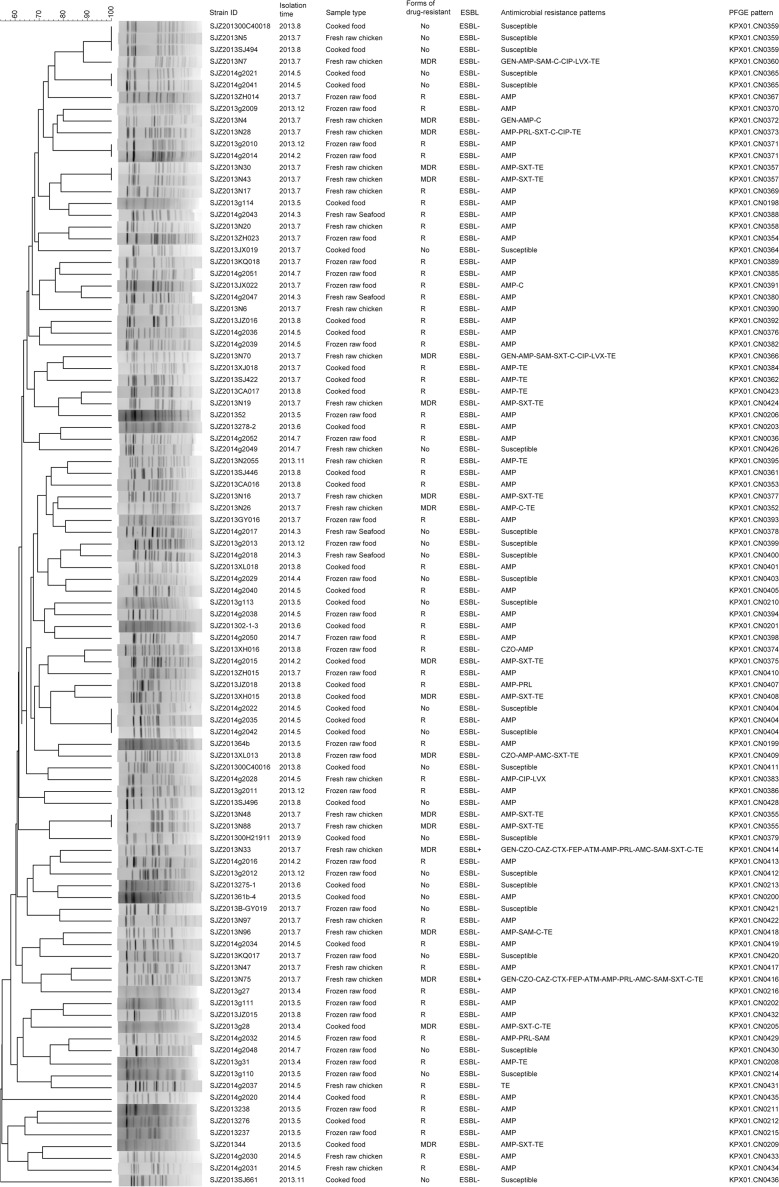
Clustering of the 99 *K*. *pneumoniae* Isolates Based on PFGE Patterns. The strain ID, isolation time, sample type, forms of drug-resistant, ESBL detection, antimicrobial resistance pattern, and PFGE pattern of each isolate are listed to the left of the patterns. In the column of “forms of drug-resistant”, “No” means not resistant to all drugs, “R” means non-susceptibility to at least 1 agent in one or two antimicrobial categories, “MDR” means non-susceptibility to at least 1 agent in 3 or more antimicrobial categories.

All the 99 isolates of *K*. *pneumoniae* were analyzed by PFGE, and 91 different PFGE patterns were obtained, with similarity values of 47.1% (Fig 1). Eighty-five (85.9%) isolates showed unique PFGE patterns. No dominant pattern was identified among these isolates. Only 6 patterns included more than 1 isolate. Two isolates of KPX01.CN0357 were isolated from fresh raw chicken samples collected from the same market at the same time; 3 isolates of KPX01.CN0404 were isolated from cooked food samples collected from the same restaurant at the same time; 2 isolates of KPX01.CN0365 were isolated from cooked food samples collected from different restaurants at the same time; and 2 isolates of KPX01.CN0355 were isolated from fresh raw chicken samples collected from different markets at the same time.

The 2 ESBL-producing isolates showed different PFGE patterns. An MLST analysis of these 2 isolates produced two unique STs (ST1651, ST1652; Table 3); neither has been previously reported.

## Discussion

Foodborne bacteria are widely studied, but research on *K*. *pneumoniae* is scarce. In a previous survey conducted in the United States, 53 (16.1%) MDR *K*. *pneumoniae* strains were isolated from 330 farm-raised frozen shrimp that were imported from Thailand to the United States [9]. In another survey focusing on fresh vegetables in Spain, 9 *K*. *pneumoniae* strains were obtained from 160 vegetables, among which 1 (0.6%) was an MDR strain [8]. The major goal of this study was to evaluate the current frequency and antimicrobial resistance of *K*. *pneumoniae* strains in fresh raw seafood, fresh raw chicken, frozen raw food and cooked food samples in China. The incidence of *K*. *pneumoniae* in these samples was 9.9%, which shows that contamination of food with *K*. *pneumoniae* is common in this region of China. Furthermore, 19 (1.9%) MDR *K*. *pneumoniae* strains were isolated in this study, representing a lower percentage than that reported by Nawaz et al. [9] but a higher percentage than that reported by Falomir et al. [8].

The highest isolation rates for *K*. *pneumoniae* and MDR *K*. *pneumoniae* strains in this study were observed for fresh raw chicken samples. Fresh raw chicken is an important reservoir of antimicrobial-resistant *K*. *pneumoniae*. A recent study focused on retail raw chicken demonstrated that β-lactamases and ESBLs were emerging and prevalent in foodborne *Salmonella* in China [38]. Profitable chicken farms demand the extensive usage of antimicrobials to inhibit infectious diseases. However, the use of antimicrobials in these ecosystems may select for antimicrobial-resistant microorganisms. Hence, chicken meat may be a reservoir of antimicrobial-resistant bacteria such as *K*. *pneumoniae*, which constitutes a public health concern.

The resistance mechanisms of *K*. *pneumoniae* include the production of β-lactamases (including ESBLs and plasmid-mediated AmpCs) and carbapenemases, the production of biological membrane formation factors, the loss of outer membrane proteins, and antimicrobial efflux [39–41]. In this study, we investigated a total of 99 *K*. *pneumoniae* isolates from food; 4 strains were ESBL, and 1 strain produced an AmpC beta-lactamase. ESBL-producing *K*. *pneumoniae* strains have been shown to have a significant impact on the treatment options and clinical outcomes of patients. Likewise, they have been shown to cause higher morbidity and mortality [42–44]. Currently, ESBLs and AmpCs are the predominant β-lactamases that mediate Gram-negative bacterial resistance to new broad-spectrum β-lactam antimicrobials. ESBLs are mainly encoded by plasmids, whereas AmpCs are mainly encoded on the chromosome. The CTX-M type is the major phenotype of domestic ESBLs; it reportedly predominates worldwide, followed by the SHV type [45–47]. Recently, Enterobacteriaceae carrying *bla*_CTX-M_-type genes were isolated from chicken in several countries [38, 48–50]. In this study, both ESBL-producing *K*. *pneumoniae* strains carried the *bla*_SHV_ gene, and one strain carried *bla*_CTX-M_ genes. One strain isolated in this study carried at least 4 ESBL-associated genes, i.e., coexisting *bla*_CTX-M-1_, *bla*_CTX-M-10_, and *bla*_SHV_. The coexistence of multiple *bla*_CTX-M_-type genes in *K*. *pneumoniae* isolates was also reported in previous studies [51,52]. The detection of ESBL-producing strains and the coexistence of several ESBL-associated genes in the same isolates pose a serious epidemiological, clinical and public health threat.

Quinolones are broad-spectrum antimicrobial agents that have been widely used in clinical medicine and for raising food-producing animals (such as chicken in China). The isolation and characterization of ciprofloxacin- and levofloxacin-resistant *K*. *pneumoniae* from fresh raw chicken samples is corresponding to that fluoroquinolones have been used in chicken farms. Among the 7 plasmid-encoded fluoroquinolone resistance-associated genes, the aac(6’)-Ib-cr enzyme, *qnrB*, *qnrA*, and *qnrS* were the most prevalent plasmid-mediated mechanisms of quinolone resistance, as previously reported [29]. Several studies have suggested that, in *K*. *pneumoniae*, DNA gyrase A is a primary target of quinolones and that *parC* alterations play a complementary role in the development of higher-level fluoroquinolone resistance [30,53]. In contrast, one study reported that hypermutation in *K*. *pneumoniae* is uncommon and does not contribute to the accumulation of *gyrA* mutations or directly to ciprofloxacin resistance [54]. Sequence analysis of the *gyrA* gene in *K*. *pneumoniae* isolates from fresh raw chicken in this study identified 3 types of *gyrA* mutations (encoding Ser83Ile, Ser83Phe, and Asp87Ala substitutions) and 1 type of *parC* mutation (encoding Ser80Ile). These point mutations were previously reported in clinical *K*. *pneumoniae* cases and may be responsible for mediating resistance to fluoroquinolones [9,51,53,55]. The amino acid substitutions at positions 83 (Ser to Phe) and 80 (Ser to Ile) in gyrase A resemble a substitution that confers fluoroquinolone resistance in *Salmonella spp*. [56,57]. Among the aminoglycosides resistance-associated genes, the *aacA4*, *aacC2*, and *aadA1* genes were detected in this study. These 3 genes were the major aminoglycosides resistance genes among clinical *K*. *pneumoniae* cases reported at a hospital in China [51], which suggests that the resistance genes in clinical strains may come from foodborne strains.

PFGE is a useful tool to reveal genotypic characteristics and to trace the reservoirs of infectious pathogens, the rates of transmission and the mechanisms of infectious diseases. The variety of different PFGE strain patterns in this study was unexpected. Except for 6 groups of isolates that had identical PFGE patterns, all other isolates showed unique PFGE patterns. Furthermore, the 2 ESBL-producing isolates showed different PFGE patterns and MLST types. These results reflected a high genetic diversity of foodborne *K*. *pneumoniae* isolates.

There were three limitations of this study. Firstly, this study had not included agricultural antimicrobial use data from the regions which supplied food to the farms, supermarkets, and restaurants from where we sampled food items. The second one was that not compared these foodborne *K*. *pneumoniae* isolates to clinical isolates from the same region. Furthermore, the use of agar SC and SS would underestimate the frequency of *K*. *pneumoniae* isolated, because these two seletive medium are designed to inhibit other microorganisms than *Salmonella* spp. and *Shigella* spp.

In conclusion, our results indicate that food, especially fresh raw chicken, is a reservoir of antimicrobial-resistant *K*. *pneumoniae*. They may have the potential to become a public health risk. Thus, our study demonstrates that improved monitoring and prevention strategies are urgently needed to better control the emergence and transmission of antimicrobial-resistant *K*. *pneumoniae* isolates.
